# The bioactivity of atraric acid as an inducer of cellular senescence in prostate cancer cells is retained by lipophilic derivatives

**DOI:** 10.1007/s00210-025-03989-0

**Published:** 2025-03-12

**Authors:** Adina Asara Baniahmad, Golnaz Atri Roozbahani, Manfred Jung, Aria Baniahmad

**Affiliations:** 1 Institute of Pharmaceutical Sciences, University of Freiburg, Albertstraße 25, 79104 Freiburg, Germany; 2 Institute of Human Genetics, Jena University Hospital, Am Klinikum 1, 07740 Jena, Germany

**Keywords:** Androgen receptor antagonist, Prostate cancer, Cellular senescence, Atraric acid

## Abstract

Esters have been described as bioactive chemical compounds. However, the presence of an ester as a functional group is often associated with hydrolytic liability. Therefore, it is often unclear whether esters serve as pro-drugs and are rather converted into bioactive drugs in cells. The small molecule atraric acid (AA) has an ester group and had been identified as the first natural androgen receptor antagonist that inhibits prostate cancer cell growth and induces cellular senescence in cancer cells. Based on the presence of the ester group, it is unclear whether AA is a prodrug being hydrolyzed to generate the bioactive compound intracellularly. Here, we addressed this issue by synthesizing novel compounds for which the ester group has been replaced by a stable isoster. To replace the methylester group of atraric acid, a ketone with a propanoyl side chain and a *N*-methoxy-*N*-methyl-amide derivative were synthesized. Functional assays suggest that both non-ester atraric acid derivatives are bioactive in inducing cellular senescence. Treatment of human prostate cancer cells with these compounds suggest that both inhibit cell growth and induce cellular senescence in a dose-dependent manner. This was observed in two different human prostate cancer cell lines that serve as model systems for androgen-sensitive and castration-resistant prostate cancer, respectively. Computational modeling suggests that these two compounds bind to the androgen receptor ligand binding domain to similar receptor residues as AA. Thus, the data suggest that replacing the ester of AA by a ketone with a propanoyl group or by the *N*-methoxy-*N*-methyl-amide group the bioactivity as an androgen receptor antagonist is retained.

**Supplementary Information:**

The online version contains supplementary material available at 10.1007/s00210-025-03989-0.

## Introduction

Bioactive compounds that act inside the cell should have a reasonable solubility in aqueous solution and be membrane permeable. In general, acids show reduced membrane permeability, whereas less charged compounds have a higher membrane permeability. Ester derivatives neutralize the positive charge of parent acids. In general, and in contrast to charged compounds, neutral molecules possess higher cell membrane permeability and thus an increase in cellular uptake. However, cleavage of esters might occur intracellularly by non-specific esterases and to produce the parent acid inside cells (Lavis 2008). Therefore, it has to be investigated in individual cases whether ester compounds are rather prodrugs that turn into the bioactive drug compounds inside the cell.

Atraric acid (AA, methyl 2,4-dihydroxy-3,6-dimethylbenzoate) is a natural compound that was isolated from the African tree *Pygeum africanum* specifically from bark extracts. AA has not a carboxylic acid group as its name might imply, but it contains two phenolic groups with acidic properties. Traditionally the bark extracts are used against prostate adenomas. Bioactivity tests with prostate cancer cells revealed that AA is an androgen receptor (AR) antagonist (Schleich et al. 2006; Papaioannou et al. 2009). AR antagonists are important therapeutics that inactivate the AR and are used clinically in prostate cancer hormone therapy.

Prostate cancer is the most diagnosed male cancer with a high mortality rate (Siegel et al. 2023). Prostate cancer is initially androgen-dependent in growth. However, eventually, the cancer becomes castration resistant but still relies on AR signaling. The tumor evolution leading to castration-resistant prostate cancer is accompanied by acquiring DNA mutations including mutations in the ligand binding domain (LBD) of the AR, amplification of AR, or non-classic activation of AR signaling that overcome inhibition by AR antagonists (Le et al. 2023; Ehsani et al. 2021).

The AR is a member of the nuclear hormone receptor family and a hormone regulated transcription factor that controls the proliferation and differentiation of the normal prostate and also prostate cancer. AR antagonists suppress cancer growth and induce cellular senescence that leads to cell cycle arrest in both androgen-dependent and castration resistant cell lines suggested as the major underlying mechanism to inhibit proliferation (Dai et al. 2017; Kokal et al. 2020; Shiota et al. 2022; Jin et al. 2024). Induction of cellular senescence was also detected in human tumor samples treated ex vivo (Hessenkemper et al. 2014). Both androgens and the clinically used second-generation AR antagonists bind to the LBD of AR. It is suggested that AR antagonists compete for androgen binding and induce a distinct receptor conformation to prevent AR activation.

Similarly, AA competes with androgens for binding to the AR (Papaioannou et al. 2009). Interestingly, AA is a non-steroidal compound and in contrast to other second-generation AR antagonists, contains only one benzene ring. Of note, AA inhibits also those AR-mutant receptors that mediate therapy resistance (Ehsani et al. 2022). To our knowledge AA has been identified as the first natural AR antagonist (Papaioannou et al. 2009). Functionally, AA inhibits the growth of androgen-dependent as well as castration-resistant prostate cancer cells. In line with this, AA induces AR-dependently the genetic program of cellular senescence in both prostate cancer types (Ehsani et al. 2022).

Since AA is an ester, it was unclear whether AA is rather a pro-drug activated intracellularly by hydrolysis leading to a dihydroxy-dimethylbenzoate compound which is a carboxylic acid. Therefore, we synthesized two novel non-ester dihydroxy-dimethylbenzoate derivatives and analyzed their biological activity in established models for both androgen-dependent and castration-resistant prostate cancer cells. Since these new derivatives show similar activity in cells, the data suggest that the ester is required for the bioactivity of AA and does not constitute a prodrug moiety. However, the ester side-chain itself is not essential and can be replaced by a much more stable ketone or an *N*-methoxy-*N*-methyl-amide side chain.

## Material and methods

### Chemical syntheses of novel compounds

All reactions were carried out in glassware. Atraric acid, *N,O*-dimethylhydroxylamine hydrochloride (98%) and HATU was purchased from BLD Pharmtech GmbH, Germany. Ethylmagnesium bromide (3 M in THF), was obtained from Sigma Aldrich and DIPEA (*N,N*-diisopropylamine) was acquired from TCI chemicals Germany Gmbh. Particularly mentioned anhydrous/dry solvents were purchased from Acros organics. Reactions were monitored by thin-layer chromatography (TLC) performed with Merck alumina plates coated with silica gel 60 F254 and analyzed under UV light (254 and 365 nm) or revealed using KMnO_4_ as staining agent, which contained 3 g of KMnO_4_, 20 g of K_2_CO_3_ in 5 mL of 5% NaOH, and 300 mL of demineralized H_2_O. The composition of the mobile phase was adjusted to the compound properties. Yields were not particularly optimized. Flash column chromatography was performed on a BiotageR Isolera Prime/One or on a BiotageR Selekt Enkel purification system using 40–60 μm pre-packed silica gel columns from BiotageR, HP-spherical 50 μm pre-packed silica gel columns from Interchim (Jumbo Pack), Sfar Silica D 60 μm. NMR spectroscopy and mass spectrometry were used for product identification. NMR spectra were acquired on a Bruker Avance 400 spectrometer (400 and 100.6 MHz for ^1^H), at a temperature of 303 K using DMSO-*d*6 as solvent (Supplementary Fig. S1). Chemical shifts (δ) are reported in ppm; multiplicity abbreviations are as follows: *s* = singlet, *d* = doublet, *t* = triplet, *q* = quartet, *m* = multiplet, coupling constant (*J*) are expressed in Hz. The ^1^H assignment resulted from COSY experiments (Supplementary Fig. S2). Mass spectra were recorded on an Advion expression CMS using an ASAPR (atmospheric solids analysis probe; aka APCI: atmospheric pressure chemical ionization) as ion source, on a Thermo Scientific Exactive mass spectrometer using electrospray ionization (ESI) as ion source or HR-MS were obtained on a Thermo Scientific Advantage. Preparative HPLC was performed for all final compounds on an Agilent 1260 Infinity II using method B at 210 nm (Supplementary Fig. S3). HPLC analysis was performed to determine the purity of all final compounds on an Agilent Technologies 1260 Infinity II system using method A diode array detector (DAD) UV detection at 210 nm.

#### Method A

XBridge**®** Shield RP18 5 μm XB-C18 100 Å 150 × 4.6 mm column and eluent A was H_2_O containing 0.05% trifluoracetic acid (TFA), and eluent B was acetonitrile. Linear gradient conditions were as follows: 0–4 min: 90:10 (A/B); 4–19 min: 90:0 → 0:100 (A/B); 19–21 min: 0:100; (A/B); 21–21.5 min: 90:10 (A/B); and 21.5–25 min: 90:10 (A/B) with a flowrate of 1.00 mL/min^–1^.

#### Method B

XBridge**®** PrepShield RP18 5 μm XB-C18 130 Å 150 × 5 mm column and eluent A was H_2_O containing 0.05% trifluoracetic acid (TFA), and eluent B was acetonitrile. Linear gradient conditions were as follows: 0–4 min: 90:10. (A/B); 4–15 min: 90:0 → 35:65 (A/B); 15–17 min: 0:100 (A/B); 17–18.5 min: 90:10 (A/B); and 18.5–19 min: 90:10 (A/B) with a flowrate of 17.10 mL/min^–1^.

### Synthesis scheme

Atraric acid (BLD Pharmatech GmbH, Germany) was used as the starting material for the synthesis of AAEB13. Following hydrolyses of the ester, the *N*-methoxy-*N*-methyl-amide was synthesized using *N,O*-dimethylhydroxylamine. In the final step, the ketone derivative was obtained by Grignard reaction with ethylmagnesium bromide.
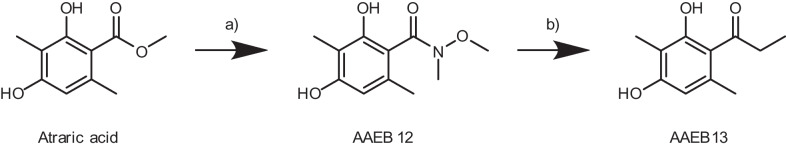


Reagents and conditions: (a) 1) KOH, rt, 16 h; 2) *N,O*-dimethylhydroxylamine, HATU, DIPEA, DCM, rt, 16 h, yield over two steps: 65%; (b) i) EtMgBr, THF, − 78° C, 4 h; ii) 0.1 M HCl, H_2_O, yield: 11%

### 2,4-Dihydroxy-3,6-dimethylbenzoic acid (AAEB-11)


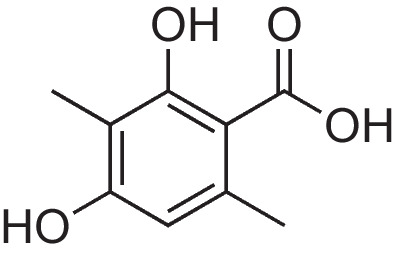


Methyl 2,4-dihydroxy-3,6-dimethylbenzoate (1.40 g, 7.06 mmol, 1 eq.) was dissolved in dem. water (14.1 mL, 0.5 M). KOH (14 mL, 1 M) was added dropwise, and the reaction was stirred for 16 h at room temperature. After the reaction was completed, an aqueous solution of HCl (1 M) was applied to acidify the mixture until the pH value was below three using a pH indicator paper. The product was extracted using dichloromethane (DCM) (25 mL × 4). The combined organic layers were dried over anhydrous Na_2_SO_4_, filtered and concentrated in vacuo. The product was a white solid and was utilized without further purification. APCI-MS (m/z): calc. for C_10_H_12_O_4_ [M + H +]: 183.06; found: 183.01.

### 2,4-Dihydroxy-*N*-methoxy-*N*,3,6-trimethylbenzamide (AAEB-12)


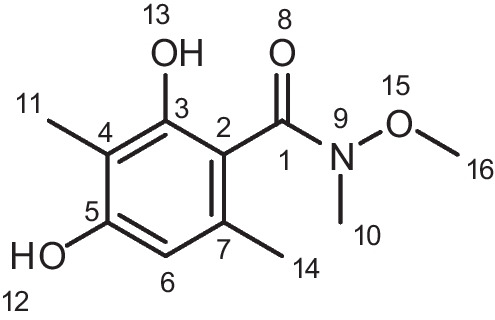


At room temperature, HATU (*O-N*, *N*, *N′*, *N′-*tetramethyluronium-hexafluorphosphate (2.00 g, 5.21 mmol, and 1.3 eq) was added to a solution of 2,4-dihydroxy-3,6-dimethylbenzoic acid (AAEB-11-crude) (900 mg and 4.0 mmol) and *N,O*-dimethylhydroxylamine (144 mg, 4.0 mmol, and 1 eq.) in dichloromethane (DCM) (40 mL and 0.1 M) and DIPEA (1.57 g, 12.0 mmol, and 3 eq.). The reaction mixture was stirred for 16 h until completion. Extraction of the product was done with DCM (25 mL × 4). The combined organic layers were dried over anhydrous Na_2_SO_4_, filtered, and concentrated in vacuo. The crude was purified with column chromatography DCM/DCM-MeOH (20%) 0–50%. The final product was a white solid. Yield: 3.02 g, 65%. ^**1**^**H NMR**: (400 MHz, DMSO-*d*_6_) δ 9.25 (s, ^1^H, O*H*), 8.41 (s, ^1^H O*H*), 6.22–6.17 (s, ^1^H, H6), 3.52 (s, 3H, 16-C*H*_*3*_), 3.12 (s, 3H, 14-C*H*_*3*_), 2.01 (s, *J* = 0.7 Hz, 3H, 10-C*H*_*3*_), 1.94 (s, 3H, 11-C*H*_*3*_). **APCI-MS** (m/z): calc. for C_11_H_15_NO_4_ [M + H +]: 226.10; found: 226.01. **Purity-HPLC** (method A): 210 nm: 88.9%.

### 1-(2,4-Dihydroxy-3,6-dimethylphenyl)propan-1-one (AAEB-13)


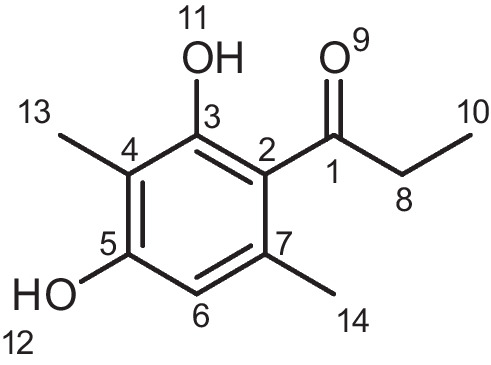


2,4-Dihydroxy-*N*-methoxy-N,3,6-trimethylbenzamide (AAB-12) (180 mg, 0.79 mmol, and 1 eq.) was cooled to − 78 °C in anhydrous THF (1.6 mL and 0.5 M) under N2 atmosphere. The solution stirred for 20 min. EtMgBr, 3 M in THF, (0.87 mmol and 1.1 eq.) was added dropwise, and the mixture was slowly warmed to room temperature for 4 h. After completion, the reaction mixture was quenched with 0.1 M HCl and extracted with EtOAc. The crude product was purified via preparative HPLC: method B. The final product was a yellow powder. ^**1**^**H NMR** (400 MHz, DMSO-*d*_6_): δ 11.56 (s, 1H, 11-O*H*), 9.88 (s, 1H, 12-O*H*), 6.24 (s, 1H, C*H*), 2.86 (q, *J* = 7.2 Hz, 2H, 8-C*H*_*2*_), 2.27 (s, 3H, 14-C*H*_*3*_), 1.93 (s, 3H, 13-C*H*_*3*_), 1.04 (t, *J* = 7.2 Hz, 3H, 10-C*H*_*3*_). **APCI-MS** (m/z): calc. for C_11_H_14_O_3_ [M + H +]: 195.23; found: 195.01; **Purity-HPLC** (methodA): 210 nm: 96.6%; yield: 17 mg.

### Cell culture and treatments

As model system for human prostate cancer, LNCaP (Protopopov et al. 2002) cells, representing androgen sensitive prostate cancer and C4-2 cells (Thalmann et al. 1994) and PC3 cells representing castration resistant prostate cancer (CRPC) were used. Cell lines were cultured at 37 °C in a humidified incubator with 5% CO_2_. LNCaP cells were grown in RPMI-1640 (Gibco Life Technologies) supplemented with 5% fetal bovine serum (FBS), 1% penicillin/streptomycin, 1% sodium pyruvate, and 2.5% HEPES 1 M (pH 7.5). C4-2 cells were cultured in DMEM medium (Gibco Life Technologies) with 20% F12 medium, 5% FBS, 1% penicillin/streptomycin, and 2.5% HEPES 1 M (pH 7.5). PC3 cells were cultured in RPMI-1640 (Gibco Life Technologies) supplemented with 10% fetal bovine serum (FBS), 1% penicillin/streptomycin, 1% sodium pyruvate, and 2.5% HEPES 1 M (pH 7.5). Various treatments, including C28 (3, 10, 30, and 100 µM), atraric acid (AA, 3, 10, 30, and 100 µM), AAEB-12 (3, 10, 30, and 100 µM), AAEB-13 (3, 10, 30, and 100 µM) or 0.1% DMSO as a solvent control, were applied to the cells.

### SA β-Gal staining assays

LNCaP (40,000) cells per well, and 30,000 C4-2 or PC3 cells per well in 6-well plates were seeded. The procedure for SA β-gal activity staining and detection followed previously described methods (Dimri et al. 1995; Roediger et al. 2014). For quantification, at least 2 areas per well, each containing a minimum of 200 cells, were counted. The percent of SA β-gal activity positively stained cells are indicated.

### Growth assays

For comparing cellular senescence and growth, 40,000 cells (LNCaP) and 30,000 (C4-2 or PC3) cells were seeded in 6-well plates with two technical replicates. Forty-eight hours after seeding, cells were treated with the indicated compounds and concentrations for 72 h prior staining with crystal violet.

For obtaining data of growth curves over 9 days in each well of 6-well plates, 13,000 cells were seeded, after 48 h of seeding cells were treated with the indicated compounds and concentrations with two wells per each. Crystal violet staining was used to detect cells. Day 0 represents data prior the start of treatment. Over the next 9 days, absorbance was measured every 3 days. The absorbance values were calculated relative to the day 0 with absorbance obtained by the solvent control DMSO.

### Analyses of mRNA expression

Reverse transcription real-time PCR was used to detect the mRNA levels as described by Ehsani et al. (2022).

### Computer modeling

We used molecular docking to explore the interactions between small molecule drugs and the androgen receptor’s ligand-binding region. The three-dimensional structure of the androgen receptor was derived from the Protein Data Bank (PDB ID 3V49). Docking simulations were performed using AutoDock Vina version 1.1.2. The first step involved the preparation of the protein. All water molecules were removed from the structure to avoid interference with the docking process. Polar hydrogens were added to the protein structure using AutoDockTools (version 1.5.7). Additionally, in the software Kollman charges were assigned. The grid box, which defined the docking search space, was then configured. The grid box’s size and coordinates were carefully chosen to contain the androgen receptor’s ligand-binding domain, ensuring that docking simulations focused on the appropriate binding location. A configuration file with all the parameters was saved. The ligand was prepared using AutoDockTools. Rotatable bonds were chosen to allow the ligand to explore several conformations during the docking procedure. This step is required to allow the ligand to adjust to the binding site. Then, the docking was performed by AutoDockVina. The resulting file ranks potential protein and ligand binding positions according to the lowest binding energy that indicates the highest binding affinity. The data were visualized with PyMOL version 2.4.0, by Schrödinger, Inc.

### Statistical analysis

Statistical analysis was performed using Graph Pad Prism 8.0 software. Data were presented as the *mean* ± *SD*, based on at least two independent experiments. Statistical significance was assessed for each experiment using either a two-tailed unpaired Students’ *t*-test (for percent staining of senescent cells and crystal violet staining) or for growth curve analyses the two-way analysis of variance (ANOVA).

## Results

**Fig. 1:**
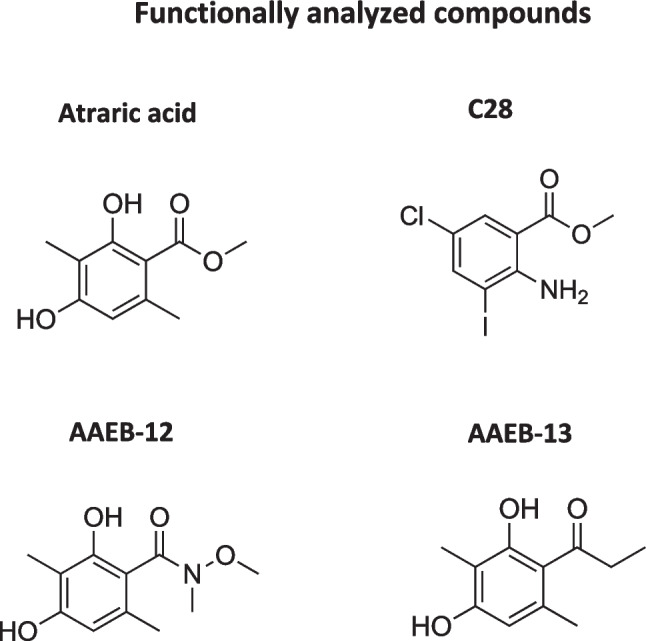
Chemical structure of AA, C28, and the two novel synthesized compounds AAEB-12 and AAEB-13

**Fig. 2:**
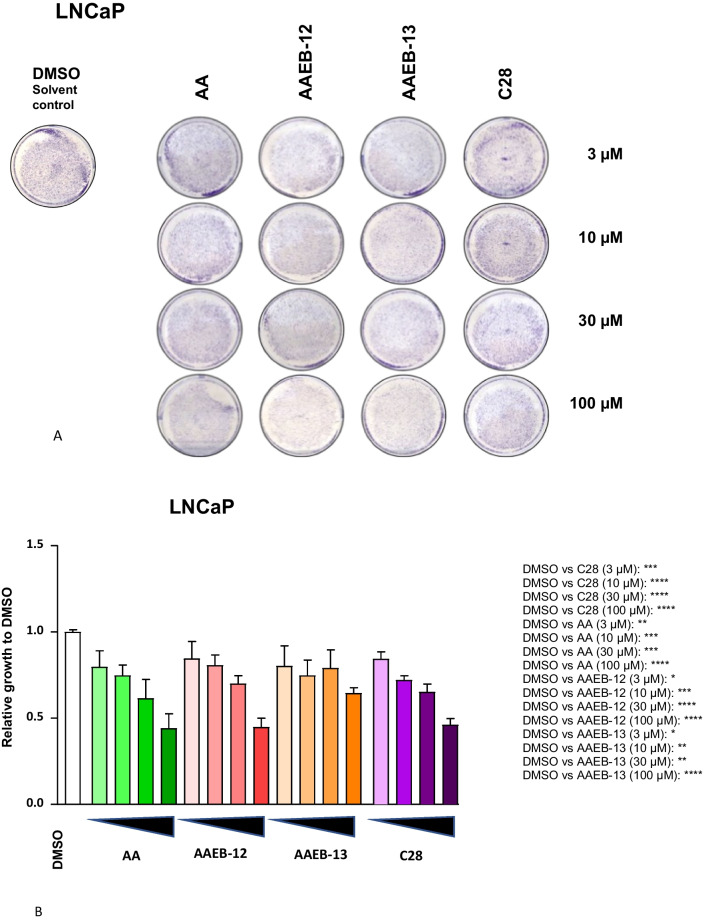
The compounds AA, C28, AAEB-12, and AAEB-13 inhibit growth of androgen-sensitive LNCaP cells. Dose responses of compounds with 3, 10, 30, and 100 µM were tested for LNCaP cell growth using crystal violet staining of cells. Cells were treated for 72 h. **A** Pictures of representative stained wells. **B** The relative growth is indicated relative to the solvent control DMSO (0.1% v/v), which is set arbitrarily as 1. Significance is calculated by the two-tailed unpaired Student’s *t*-test and is indicated with *P* value < 0.0001 = ****, < 0.001 = ***, < 0.01 = **, < 0.05 = *

**Fig. 3:**
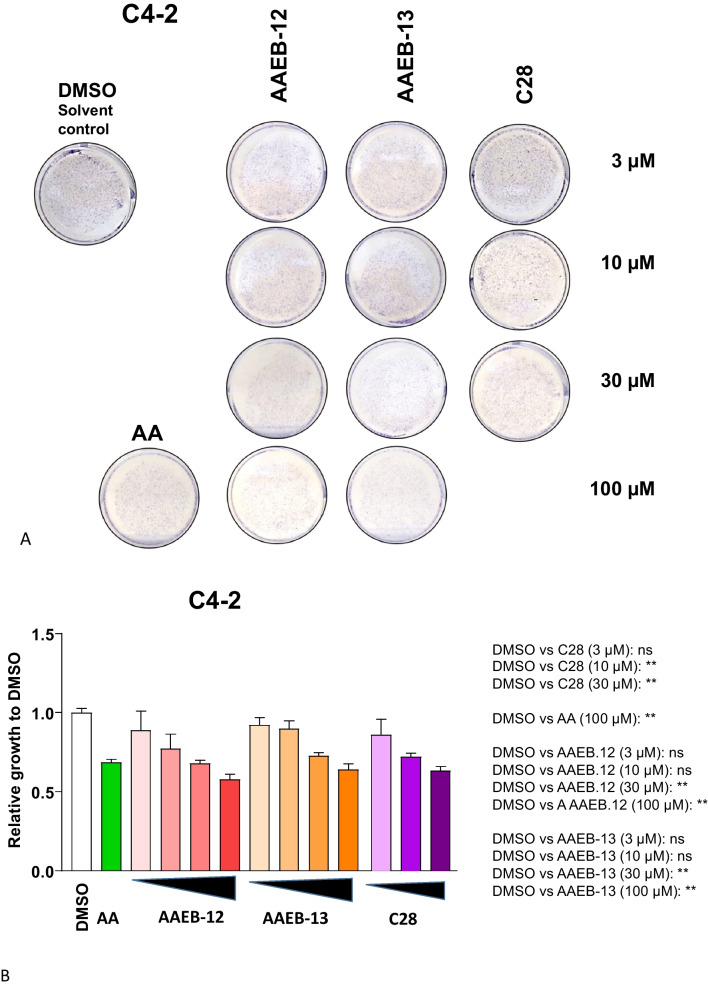
AA, C28, AAEB-12, and AAEB-13 inhibit growth of castration-resistant C4-2 cells. Similar to Fig. 2, dose responses of compounds with 3, 10, 30, and 100 µM were analyzed by treating C4-2 cells for 72 h. **A** Cell growth was measured by crystal violet staining of cells. **B** Summary of data with significance calculated by the two-tailed unpaired Student’s *t*-test. *ns* = non-significant. *P* value < 0.01 = **, < 0.05 = *

Replacing the ester group of AA (Fig. 1) the following derivatives were chemically synthesized: 2,4-dihydroxy-*N*-methoxy-*N*,3,6-trimethylbenzamide (AAEB-12) and 1-(2,4-dihydroxy-3,6-dimethylphenyl) propan-1-one (AAEB-13). In addition, the recently characterized AR antagonist compound C28 (Roell et al. 2019; Fenner 2019; Fig. 1), that is also composed of only one benzene ring, was included in the functional assays. Growth analyses were performed with the human prostate cancer model cell lines representing the androgen-sensitive LNCaP and the castration-resistant C4-2 cell lines (Figs. 2 and 3). Dose response was analyzed by treating both cell lines with increasing concentrations of compounds for 72 h. Using crystal violet staining the cell growth was analyzed and using the established marker for cellular senescence, the senescence-associated beta galactosidase activity (SA β-gal) assay was detected by X-Gal staining. The data suggest that C28, AA, and AAEB-12 exhibit a similar bioactivity in inhibiting cell growth in LNCaP cells, whereas AAEB-13 showed a weaker cellular response (Fig. 2A, B and Table 1).

**Table 1:** Inhibition of growth at day 3 IC50

Compounds	IC50-LNCaP (μM)	IC50-C4-2 (μM)
C28	88	68
AA	76	-
AA12-EB	90	126
AA13-EB	180	200

Interestingly, in the CRPC C4-2 cells treatment with C28, AA, AAEB-12 and AAEB-13 inhibited cell growth similarly at the highest concentrations (Fig. 3A, B) with a slightly higher IC_50_ for AAEB-12 and AAEB-13 compared to LNCaP cells, which may be due to the castration-resistance and more aggressive form of PCa cells.

**Fig. 4:**
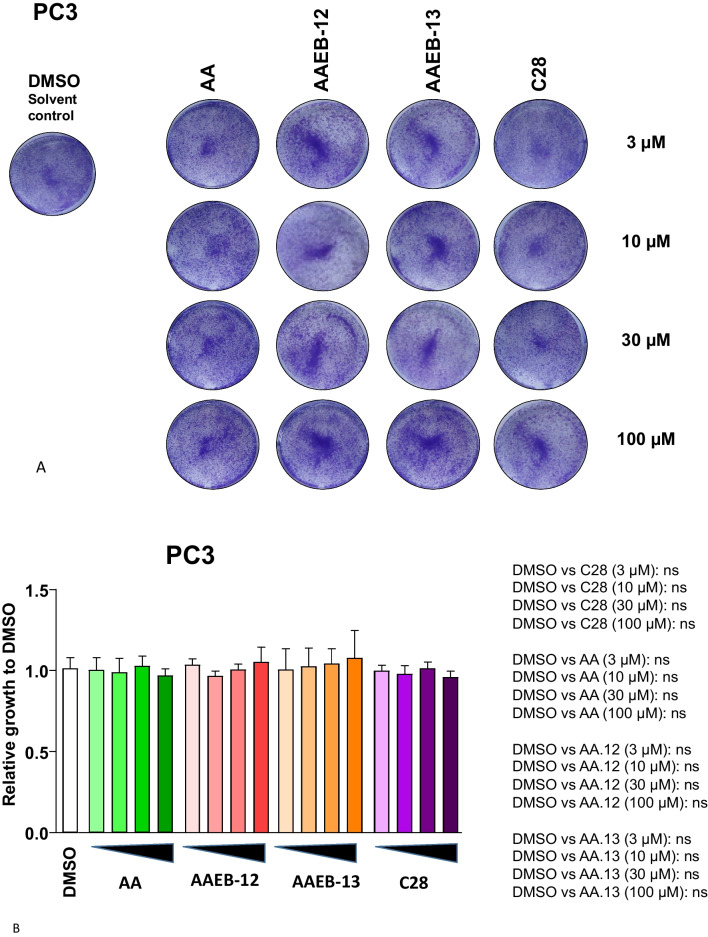
AA, C28, AAEB-12, and AAEB-13 lack inhibition of growth of castration-resistant PC3 cells. Similar to Fig. 2, dose responses of compounds with 3, 10, 30, and 100 µM were analyzed by treating PC3 cells for 72 h. **A** Cell growth was measured by crystal violet staining of cells. **B** Summary of data with significance calculated by the two-tailed unpaired Student’s *t*-test. *ns* = non-significant

In addition, the AR-negative CRPC PC3 cells were employed (Fig. 4A, B). Treatment with the compounds did not lead to changes in cell proliferation suggesting that the compounds used act in an AR-specific manner. On one hand the AR-depend bioactivity a benefit that may reduce side-effects and on the other hand, it limits potential treatment to AR expressing prostate cancer cells.

**Fig. 5:**
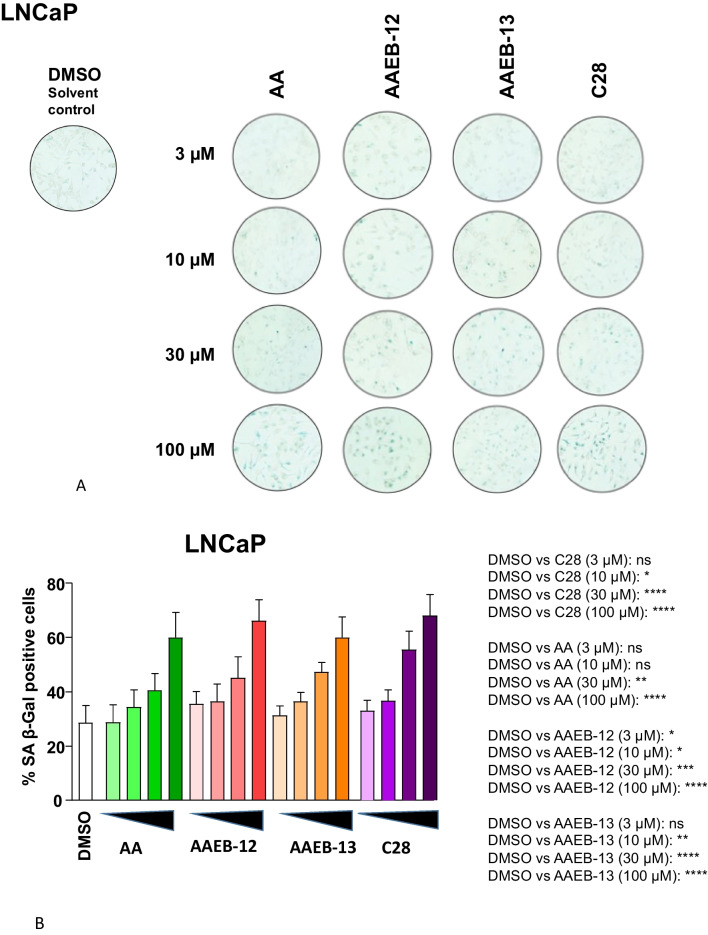
AA, C28, AAEB-12, and AAEB-13 induce the senescence-associated beta-galactosidase activity in LNCaP cells. LNCaP cells were treated with the indicated compounds by increasing concentrations as indicated in Fig. 2 for 72 h prior to the detection of the cellular senescence marker SA β-Gal activity. DMSO serves as solvent control. **A** Pictures of wells with stained cells. Two to 4 areas of each well with 200 cells each were counted. **B** The numbers indicate the percent of positively stained cells. The significance is calculated by the two-tailed unpaired Student’s *t*-test. *ns* = non-significant, *P* value < 0.0001 = ****, < 0.001 = ***, < 0.01 = **, < 0.05 = *

**Fig. 6:**
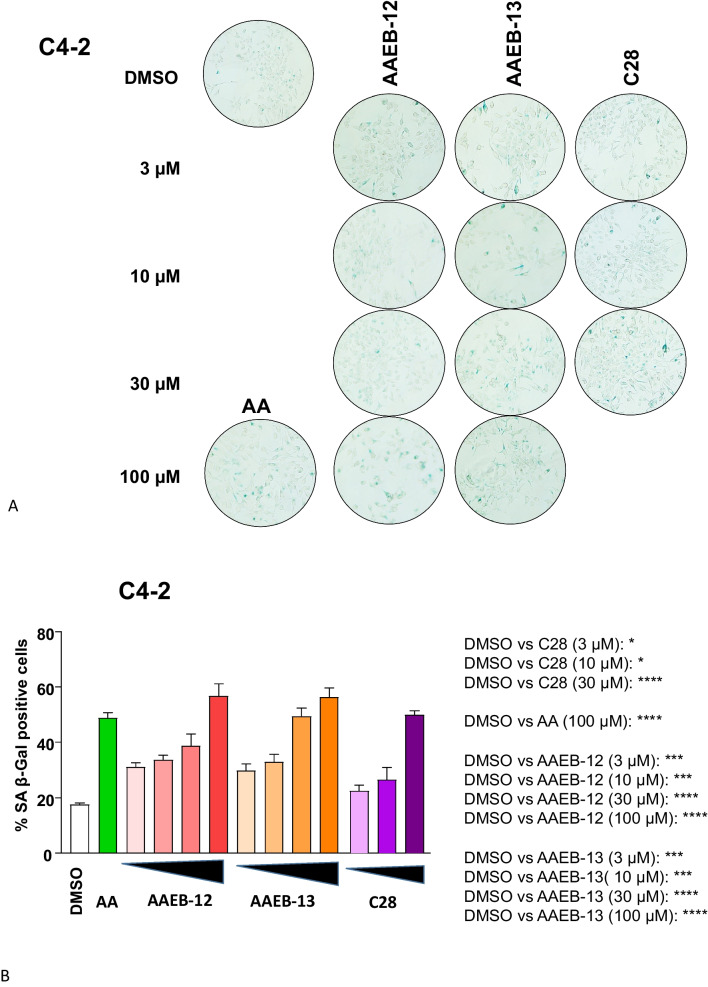
AA, C28, AAEB-12, and AAEB-13 induce the senescence-associated beta-galactosidase activity in C4-2 cells. Dose response of induction of SA β-Gal activity in C4-2 cells that were treated with the indicated compounds by increasing concentrations as indicated in Fig. 2 for 72 h prior to the detection of the cellular senescence marker SA β-Gal activity. DMSO serves as solvent control. **A** Pictures of wells with stained cells. Two to 5 areas of each well with 200 cells each were counted. **B** The numbers indicate the percent of positively stained cells. The significance is calculated by the two-tailed unpaired Student’s *t*-test. *ns* = non-significant, *P* value < 0.0001 = ****, < 0.001 = ***, < 0.01 = **, < 0.05 = *

**Fig. 7:**
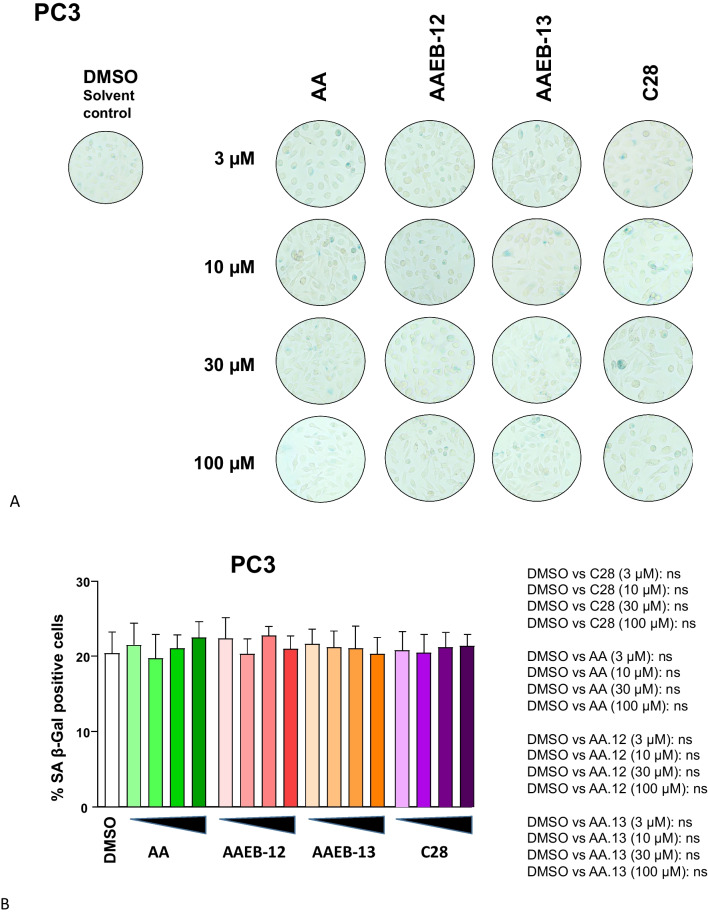
AA, C28, AAEB-12 and AAEB-13 treatment lack induction of senescence-associated beta-galactosidase activity in PC3 cells. Dose response of induction of SA β-Gal activity in PC3 cells that were treated with the indicated compounds by increasing concentrations as indicated in Fig. 2 for 72 h prior the detection of the cellular senescence marker SA β-Gal activity. DMSO serves as solvent control. **A** Pictures of wells with stained cells. Two to 5 areas of each well with each 200 cells were counted. **B** The numbers indicate the percent of positively stained cells. The significance is calculated by the two-tailed unpaired Student’s *t*-test. *ns* = non-significant

An important feature of AR antagonists is the induction of cellular senescence observed with second-generation AR antagonists (Kallenbach et al. 2022; Gupta et al. 2020). In line with growth inhibition, increasing concentrations of each of the compounds enhance cellular senescence levels in both LNCaP (Fig. 5A, B) and C4-2 cells (Fig. 6A, B). Of note, a concentration dependent induction of cellular senescence has been shown previously (Ehsani et al. 2022). Also according to the growth inhibition, the overall induction of cellular senescence is weaker in the CRPC cells C4-2 compared to LNCaP cells. Using the AR-negative PC3 cells, no significant changes in the cellular senescence levels were observed by treating the cells with the compounds, further supporting the indication that the compounds act in an AR-dependent manner (Fig. 7A, B).

The expression of CDKN2A encoding the cell cycle inhibitor p16 has been shown to correlate with the induction of cellular senescence in prostate cancer cells (Roediger et al. 2014). The obtained data suggest that the increase of *CDKN2A* levels of treated cells correlate with induction of cellular senescence (Supplementary Fig. S5), whereas in PC3 that lack induction of cellular senescence, no significant changes of *CDKN2A* expression was detected (Supplementary Fig. S6), confirming AR-dependent changes of cellular senescence by the compounds and supporting the notion that the bioactivity as AR antagonist is retained by both AA-derivatives.

**Fig. 8:**
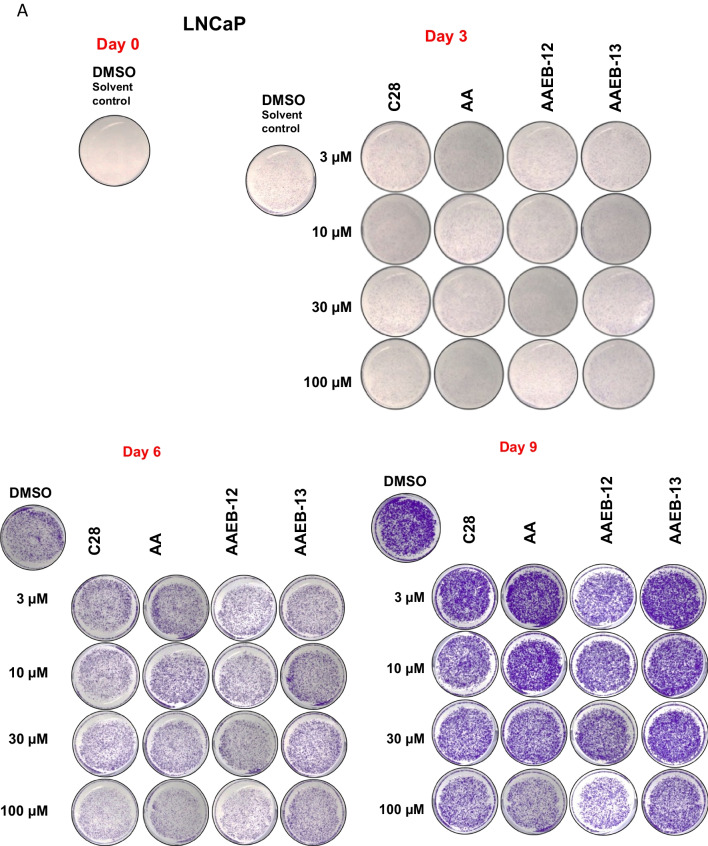

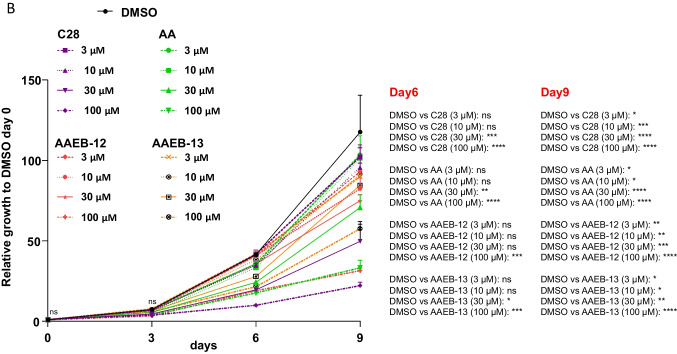
Growth inhibitory response of LNCaP cells by long-term treatment of 9 days with AA, C28, AAEB-12, and AAEB-13 indicates that AAEB-12 has a similar potency as AA. LNCaP cells were treated for 9 days refreshing the medium and compounds every 72 h. Crystal violet staining was used for cell staining. **A** Pictures of representative stained wells. **B** The relative growth is indicated relative to the solvent control DMSO (0.1% v/v), which is set arbitrarily as 1. Significance is calculated by two-way analysis of variance (ANOVA) and is indicated with *P* value < 0.0001 = ****, < 0.001 = ***, < 0.01 = **, < 0.05 = *, *ns* = non-significant

Analyzing the treatment time for a longer period using 9 days, the analysis suggests that besides C28, AA and AAEB-12 are most potently inhibiting cell growth at day 9 (Fig. 8A, B; Table 2). AAEB-13 inhibits cell growth less effectively at 100 µM as compared to AA with 100 µM. Interestingly, the IC_50_ at 9 days treatment is lower compared to day 3, which may be due to the induction of the cellular program of cell senescence that requires time being induced at a maximum not until day 3 of treatment (Roediger et al. 2014).

**Table 2:** Inhibition of growth at day 9 IC50

Compounds	IC50-LNCaP (μM)
C28	23
AA	49
AA12-EB	56
AA13-EB	96

**Fig. 9:**
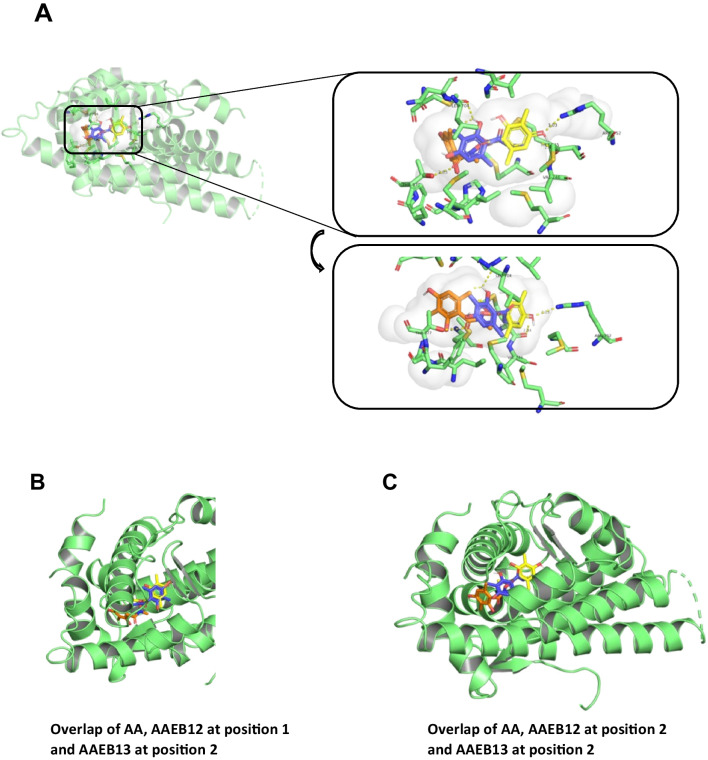

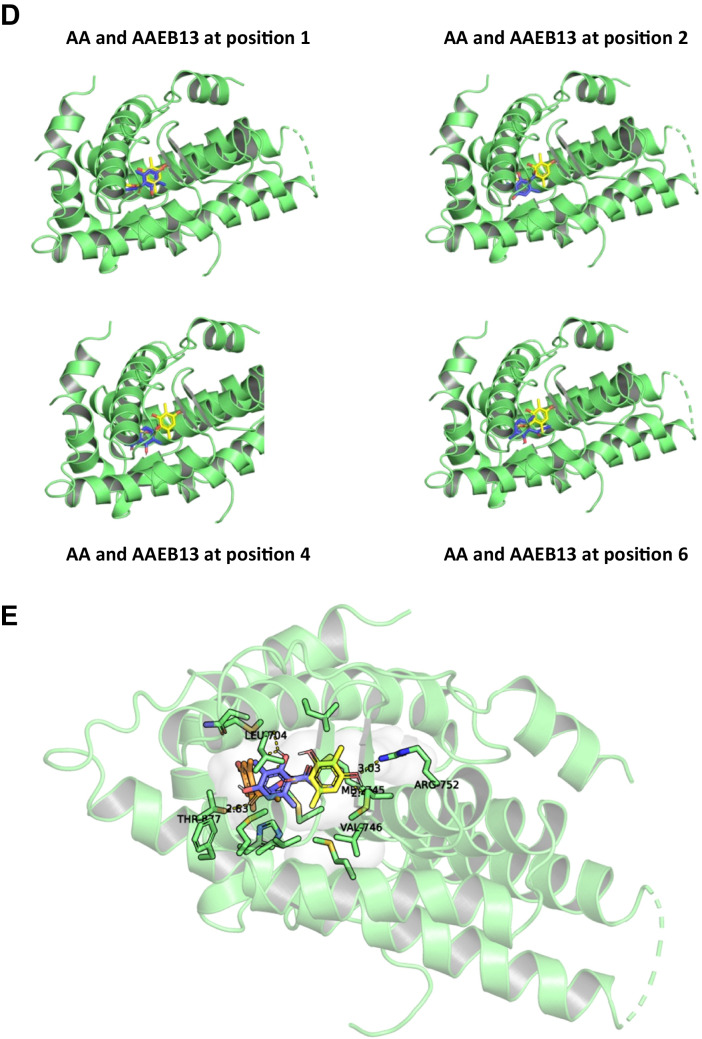
Docking modeling suggests binding of AA and AAEB-12 and AAEB-13 to similar sites within the ligand pocket domain of the AR. **A** On the left, the protein model of AR (PDB: 3V49) is displayed in green. On the right, a zoomed-in view of the binding pocket is shown, highlighting interactions with the ligands. **B** An overlap of the docked positions of AA (yellow), AAEB12 (orange, in docking position 2), and AAEB13 (blue, in docking position 1) is depicted. **C** A separate image shows AAEB13 bound to AR in position 2. **D** The binding of AA to AR is displayed, alongside various binding positions of AAEB13 (positions 1, 2, 4, and 6), indicating potential multiple binding modes. **E** A closer view of the binding pocket is shown, detailing the amino acids involved in binding the compounds. This structured breakdown of the images and docking positions helps in better understanding the interactions and binding modes of the ligands with the AR protein

Computer modeling of binding of ***AAEB-12*** and −13 was compared to that of AA using AutoDock Vina (version 1.1.2) software and the crystal structure of the AR LBD from protein data bank: 3V49 (Nique et al 2012). The obtained free energy level suggests various binding options (Table 3). The data were visualized by PyMOL (version 2.4.0). The interactions of AA, AAEB-12, and AAEB-13 with the AR were predicted to involve the following binding residues. AA forms a hydrogen bond with arginine-752. AAEB-13, predicted in position two, binds to leucine-704 and an intramolecular water molecule. AAEB-12 binds to the threonine-877 via a hydrogen bond (Fig. 9 and supplemental Fig. S7). This finding could be interesting that minor modifications of the compounds are predicted to potentially interact within a similar region within the LBD of AR with other residues and may provide insights into the interaction of AR antagonists with the receptor. Thus, the results suggest a similar location of these compounds within the ligand pocket of the AR to act as AR antagonists.

**Table 3:** Positions and affinity of AA and AAEB-compounds with AR LBD

	Affinity (kcal/mol)
AA docking sites	
1	− 6
3	− 5.9
5	− 5.7
AAEB 13 docking sites	
1	− 6.3
2	− 6.1
4	− 5.9
6	− 5.7
7	− 5.7
AAEB 12 docking sites	
2	− 6.47

## Discussion

It emerged in recent years that AR antagonists do not inactivate the AR signaling but induce the cellular pathway of cellular senescence, which is a potent cell cycle inhibitory pathway and may account for the growth inhibition by AR antagonists. Cellular senescence is induced by the second generation AR antagonists enzalutamide and darolutamide (Gupta et al. 2020). Similarly, cellular senescence can be induced by the compound C28 as structurally different AR antagonist (Roell et al. 2019). AA induces cellular senescence in LNCaP and C4-2 cells in a concentration-dependent manner (Hessenkemper et al. 2014; Ehsani et al. 2022). Also, induction of cellular senescence by AA was observed in prostate cancer tissues treated ex vivo with AA. However, it was unclear whether AA is a pro-drug that will be converted to the free acid inside the cells enzymatically by hydrolysis. The free acid of AA, however, is not bioactive (Papaioannou et al. 2013). However, the lack of bioactivity of the free acid of AA may be due to poor cellular permeability. Therefore, we generated AA derivatives that lack the ester group but retain a lipophilic side chain. It was replaced by the *N*-methoxy-*N*-methyl-amide group (AAEB-12) and also by a ketone (AAEB-13) with a similar lipophilicity as AA (Supplemental Fig. S4).

The goal of this study was to analyze whether these non-ester compounds act similar to AA which indicates that AA is not necessarily a prodrug being hydrolyzed to generate the bioactive compound. Xenografted mice with human PCa tumors that have been treated with AA show no obvious sign of toxicity in mice (Ehsani et al. 2022). The in vivo toxicity of our new compounds is yet to be determined.

Importantly, functional assays reveal that both compounds exhibit a similar bioactivity in reducing growth and induce cellular senescence in PCa cells. Furthermore, similar to AA, the analyzed bioactivity of both compounds is AR-dependent. Therefore, we assume that AA is not a pro-drug to be hydrolyzed but the actual drug interacting with the AR. This was confirmed by computer modeling that indicates a similar binding location within the AR ligand pocket of the AR ligand binding domain. These findings indicate that ester compounds may act as bioactive drugs and may not necessarily be considered in general per se as pro-drugs. Further, the data suggest that two novel compounds were identified that possess a similar bioactivity as compared to AA in modulating AR signaling. Compared to the chemical platforms of the first- and second-generation AR antagonists such as bicalutamide, enzalutamide, and darolutamide, it suggests that smaller chemical scaffolds with only one benzene ring can also act as AR antagonists.

## Supplementary Information

Below is the link to the electronic supplementary material.

Supplementary file1 (DOCX 3390 KB)

## Data Availability

All source data for this work (or generated in this study) are available upon reasonable request.
