# Supplementary material for: The bioactivity of atraric acid as an inducer of cellular senescence in prostate cancer cells is retained by lipophilic derivatives
Source: Naunyn Schmiedebergs Arch Pharmacol. 2025 Mar 12;398(9):11803–19. doi: 10.1007/s00210-025-03989-0 (PMC12449410; doi:10.1007/s00210-025-03989-0)
Supplement: Supplementary file 1 — Supplementary file1 (DOCX 3390 KB) [file 210_2025_3989_MOESM1_ESM.docx]

**Supplemental figures**
